# NeuroRDF: semantic integration of highly curated data to prioritize biomarker candidates in Alzheimer's disease

**DOI:** 10.1186/s13326-016-0079-8

**Published:** 2016-07-08

**Authors:** Anandhi Iyappan, Shweta Bagewadi Kawalia, Tamara Raschka, Martin Hofmann-Apitius, Philipp Senger

**Affiliations:** Department of Bioinformatics, Fraunhofer Institute for Algorithms and Scientific Computing (SCAI), Schloss Birlinghoven, 53754 Sankt Augustin, Germany; Bonn-Aachen International Center for Information Technology, Rheinische Friedrich-Wilhelms-Universität Bonn, 53113 Bonn, Germany; University of Applied Sciences Koblenz, RheinAhrCampus, Joseph-Rovan-Allee 2, 53424 Remagen, Germany

**Keywords:** RDF, Semantic web, Data integration, Data curation, Data harmonization, Disease modeling, Neurodegenerative diseases, Alzheimer's disease

## Abstract

**Background:**

Neurodegenerative diseases are incurable and debilitating indications with huge social and economic impact, where much is still to be learnt about the underlying molecular events. Mechanistic disease models could offer a knowledge framework to help decipher the complex interactions that occur at molecular and cellular levels. This motivates the need for the development of an approach integrating highly curated and heterogeneous data into a disease model of different regulatory data layers. Although several disease models exist, they often do not consider the quality of underlying data. Moreover, even with the current advancements in semantic web technology, we still do not have cure for complex diseases like Alzheimer’s disease. One of the key reasons accountable for this could be the increasing gap between generated data and the derived knowledge.

**Results:**

In this paper, we describe an approach, called as *NeuroRDF*, to develop an integrative framework for modeling curated knowledge in the area of complex neurodegenerative diseases. The core of this strategy lies in the usage of well curated and context specific data for integration into one single semantic web-based framework, RDF. This increases the probability of the derived knowledge to be novel and reliable in a specific disease context. This infrastructure integrates highly curated data from databases (Bind, IntAct, etc.), literature (PubMed), and gene expression resources (such as GEO and ArrayExpress). We illustrate the effectiveness of our approach by asking real-world biomedical questions that link these resources to prioritize the plausible biomarker candidates. Among the 13 prioritized candidate genes, we identified MIF to be a potential emerging candidate due to its role as a pro-inflammatory cytokine. We additionally report on the effort and challenges faced during generation of such an indication-specific knowledge base comprising of curated and quality-controlled data.

**Conclusion:**

Although many alternative approaches have been proposed and practiced for modeling diseases, the semantic web technology is a flexible and well established solution for harmonized aggregation. The benefit of this work, to use high quality and context specific data, becomes apparent in speculating previously unattended biomarker candidates around a well-known mechanism, further leveraged for experimental investigations.

**Electronic supplementary material:**

The online version of this article (doi:10.1186/s13326-016-0079-8) contains supplementary material, which is available to authorized users.

## Background

Alzheimer's disease (AD), the most prominent neurodegenerative disease (NDD), has become a global threat to the aging society, affecting nearly 115 million people by 2050 [1]. The imperfect understanding of the AD etiology has created a large gap in translating the pre-clinical findings into clinical trials dominantly observed in high drug attrition rates [2]. Early diagnosis and preventive interventions could facilitate substantial reduction in the number of affected cases to 9 million by 2050 [3, 4]. Particularly, reliable biological markers of disease and disease progression could assist in early diagnosis and treatments catered to the patient [5]. In this direction, considerable global research efforts have been dedicated to investigate molecular players underlying AD pathogenic events, contributing to an ever-growing wealth of disparate data. Refinement of this information into actionable knowledge representations requires a good interoperable and formalized framework, capable of inferring potential biomarkers across different facets of the molecular physiology. Additionally, in silico disease models that integrate complementary data from various resources are capable of recapitulating key mechanisms for a given condition [6–8].

Among others, most widely used data integration strategies include data warehousing (e. g., Pathway Commons [9]), data centralization (e. g., UniProt [10], IntAct [11]), and federated databases (e. g., BioMart [12]). An example of a data integration framework is tranSMART [13], which consists of a data warehouse covering various types of data and related data mining applications required for translational research and biomarker discovery workflows. Such a harmonized aggregation of heterogeneous data sources facilitates interpretation over a large knowledge space [14].

However, one fundamental challenge with most of these integration approaches is to cope with the variability and heterogeneity in content, language, and formats of incoming data from different source repositories. Moreover, regular updates of these data resources are necessary to keep up with newly added information and to avoid incompleteness. The inaccessibility to the integrated data resources, due to altered database structure or change in the naming conventions is unavoidable [15]. Semantic web technologies have overcome the above described challenges up to an extent by revolutionizing the lossless exchange of data and formalizing the data format into a computable knowledge [16], calling it “smart data" [17]. The capability of using rich formal descriptions for data and its standardized mapping allows complex querying in a more efficient way without information loss.

Resource Description Framework (RDF) is the World Wide Web Consortium (W3C) proposed standard for semantic integration and modeling of data. RDF uses the syntax of Extensible Markup Language (XML) and imposes structural constraints to represent the meta-data as a set of triples containing directed edges. One big advantage lies in the usage of common namespaces across the different data domains encoded as Unified Resource Identifiers (URIs). Initiatives such as Identifiers.org [18] provide persistent official identifiers in the biomedical domain, allowing sustained interlinking between distinct data resources. This allows high levels of seamless interoperability between data sources and the capability to access and map against additional related data unambiguously, called data federation. On the contrary, large efforts are still needed during an initial definition of the ontologies to build the schema for data representation.

### Semantics in life sciences

The idea of semantic web prevails in various domains, including life sciences. Recently, "The Monarch Initiative" [19] has taken the semantic route to enable reasoning over genotype-phenotype equivalence within and across species. They leverage on ontologies to link external curated data resources for generating new hypothesis and prioritizing candidates/variants based on the phenotypic similarity. Stevens et al. [20] launched TAMBIS, multi-data application tool, which allows biologists to formulate complex molecular biology questions to databases such as Swiss-Prot [21], Enzyme [22], CATH [23], BLAST [24], and Prosite [25] through well-defined semantics.

Among the early users of RDF, Lindemann et al. [26] applied it to centralize and flexibly access the heterogeneous and varying quality of medical data obtained from several clinical partners. The importance of semantic mining in the life science domain was brought to limelight by the Bio2RDF project [27], which demonstrated the possibility of querying life science knowledgebases by linking public bioinformatics databases and providing public SPARQL endpoints. Subsequently, Linking Open Drug Data (LODD) [16] demonstrated linking drug data information from DrugBank [28] and clinical trials resources. Chem2Bio2RDF [29] demonstrates the potential usage of the above two mentioned RDF repositories in the field of chemoinformatics.

Observing the immense advantage of linked open data, several major publicly available life science databases such as UniProt, DisGeNet [30], Protein Data Bank Japan (PDBj) [17], and EBI resources such as Gene Expression Atlas [31], ChEMBL [32], BioModels [33], Reactome [34], and BioSamples [35], have made their data available in the form of RDF. Thus, the RDF platform has been increasingly adopted as a standard for data exchange. Amidst prime users of RDF in elucidating disease pathophysiology, Shin et al. [36] demonstrated systematic querying of linked experimental data to explore the effect of genes that are regulated by volatile organic compounds in human blood. Qu et al. [6] showed semantic framework capability in drug re-purposing by proposing Tamoxifen, an FDA approved drug for Breast Cancer, as a candidate drug for Systemic Lupus Erythematosus. The above reported association has already been tested in mice by Sthoeger et al. [37], showing a leverage of semantic web in a real world scenario. Furthermore, Willighagen et al. [38] presented the linkage of several RDF technologies in molecular chemoinformatics and proteochemometrics.

To our knowledge, there has been very limited application of semantic web approaches to the research of neurodegenerative diseases. Linked Brain Data (LBD) [39] is an upcoming initiative which focusses on understanding the brain functionality by integrating resources such as genomic, proteomic, anatomical and biochemical resources with respect to neuroscience. Using such a multi-level knowledgebase, they aim to understand the association between cognitive functions and brain diseases. Lam et al. [40] made the first attempt to develop an e-Neuroscience data integration framework, AlzPharm [41]. They extracted AD-related drug information from BrainPharm [42] to be further integrated with manually inferred hypotheses from the scientific literature and published articles (SWAN [43]). They demonstrated the usage of such a model by clustering AD drugs based on their molecular targets and to filter publications (claims and hypotheses) specific to Donepezil effect on treatment of AD. Although AlzPharm made use of manually inferred hypothesis, they lack the validation of their findings with experimental data such as gene expression and pathways.

### Motivation

Despite the current advancements in semantic web technology, we still do not have cure for complex diseases like AD. One of the key reasons accountable for this could be the increasing gap between generated data and the derived knowledge. In order to increase the probability of the derived knowledge to be novel, data quality and data reliability is highly desirable. Moreover, the contextual specificity of the data is of paramount importance.

Compared to relational database management system (RDBMS) technologies, in RDF the relations have explicit meaning (expressiveness) in a given context and are directly accessible; allowing the user to extract meaningful knowledge from the data as opposed to an unstated structured data. In addition, RDF structures are more adaptive and flexible, allowing fluidity in the data relationships. This overcomes the fragility of RDBMS; where if the underlying representation of the keys and flat table changes, the tentacled connections are lost. Moreover, triples from RDF can be transformed into RDBMS structure and vice-versa. One another advantage of RDF is its graph representation that enables us to better explore relations through network topological characteristics such as relatedness, network perturbation, centrality, influence, etc. The usage of automated reasoners have largely been beneficial to understand the semantics and to expand the associated relations [44].

In this paper we propose *NeuroRDF*, an approach harnessing the potential of RDF as a framework for modeling neurodegenerative diseases to enable a close, biologically sensitive integration of well curated, complementary, and multi-faceted data. The fundamental principle of this strategy is to take advantage of semantics to develop a context specific, multi-layered in silico disease model, represented as a formalized, and computationally processable domain knowledge. A fine-grained analysis of the metadata from various data resources empowers the user to ask more focused questions around a hypothesized pathomechanism involving previously neglected or hidden candidates, further leveraged for experimental investigations. Considerable efforts have been invested to process and manually curate huge amounts of data that is required to build such a knowledge base around a specific indication. This includes for example the in-depth assessment of the respective phenotype, the type of tissue used in an experiment, and information around the donor of the tissue like gender, age, and possible comorbidities. Querying such a highly curated and focused knowledgebase increases the chances of unraveling novel hypothesis, which could have been lost over time or pave way to newly emerging knowledge.

We used SPARQL to traverse each of these knowledge graphs (derived from distinct resources) in an integrative manner, allowing highly disease specific analysis of the underlying data. Using this approach, we demonstrate an example on how to prioritize novel candidates in AD mechanism.

## Methods

**Fig. 1:**
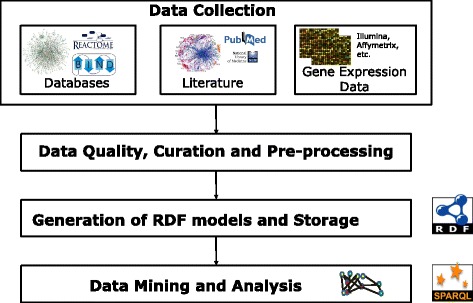
Overall workflow of NeuroRDF. The workflow illustrates the collection of data from various resources such as databases, and literature, followed by steps taken to pre-process and prune the collected data. These high-quality data are represented semantically as RDF models and stored in a triplestore. The stored knowledge can later be queried for biologically interesting questions

The developed generic semantic web-based workflow integrating heterogeneous data resources is outlined in Fig. 1. This multi-layered model integrates data from various public resources such as databases, literature, and gene expression information. The harmonization of heterogeneous data to build RDF models was achieved by using several data/file parsers. The workflow also includes a pre-processing step to monitor the quality of each incoming data type for specificity.

### Data collection and resources

This subsection depicts briefly the different data resources integrated into the *NeuroRDF*.

#### Database-derived interactions for healthy brain

A closer look into the healthy human brain interactions could improve identification of the dysregulated mechanisms which further surges the plausibility of identifying AD drugs in clinical trails [45, 46]. However, the mainstream AD research is biased towards the well known disrupted events such as APP, and tau rather than recognizing their role in normal brain functions [47].

Several publicly available databases provide protein-protein interactions (PPIs) and microRNA-target interactions (MTIs), which can be derived using multiple sources and methodologies. For instance, Human Protein Reference Database [48], Molecular INTeraction database [49], and miRTarBase [50] focus on experimentally verified interactions that are manually mined from literature by expert biologists. In addition to literature-derived information, Biomolecular Interaction Network Database [51] centralizes interactions from high-throughput technologies. Few other databases such as STRING [52], and miRWalk [53] also provide predicted interactions. However, none of these databases mine interactions specific to a given context (for example AD pathology or normal physiology).

A lot of published healthy state PPIs are not directly measured in human cells but in artificial conditions such as human cell lines, human genes transfected into yeast cells, etc., missing out on the biological plausibility in humans and context specificity [54]. Hence, considerable effort by Bossi and Lehner [55] was invested to verify the tissue specificity of PPI interactions from 21 databases (including a few above mentioned) using human gene expression data. Furthermore, this additional action to ensure validity of the interactions in normal state aids improved prediction of genes in disease state [56]. In that direction, our group has extracted a subset of these experimentally confirmed PPIs belonging to healthy brain physiology [57]. Currently, the healthy brain PPI network contains 7,192 genes and 45,001 PPIs.

#### Extracting AD-specific interactions from literature

The bridging factor between researchers and scientific accomplishments are published as texts, warehoused in large repositories like PubMed [58]. These biomedical articles are the major information source of functional factors such as proteins, genes, microRNAs (miRNAs), etc. However, their functional descriptions are scattered as unstructured text in literature [59]. Text-mining methods could help us mine these articles and retrieve the associated relations/evidence for a given context. Since proteins are the chief players in almost all biological processes and miRNAs have been established in the last decade as important regulators of gene expression, we focus our current research on MTIs and PPIs.

In order to harvest AD-specific knowledge from the literature, we used our in-house state-of-the-art named entity recognition (NER) system ProMiner [60] and the semantic search engine SCAIView [61]. Identification of genes/proteins and disease mentions was accomplished using dictionaries. The disease dictionary was built using MeSH [62], MedDRA [63], and Allie [64] databases. Currently, it contains 4,729 concepts and 64,776 synonyms [65], which are normalized to MeSH names. Human Genes/Proteins dictionary [60] was compiled from three different resources: SwissProt, EntrezGene [66], and HGNC [67]. Currently, this dictionary consists of 36,312 entries and 515,191 synonyms. All the identified gene/protein names were normalized to HUGO gene symbols for maintaining homogeneity across all data resources and also for future comparisons and visualizations.

To identify MTIs from MEDLINE abstracts, we applied our previously developed approach [65]. Here we extracted novel miRNA mentions using a regular expression. These mentions were normalized to miRBase database identifiers [68]. In addition, relation dictionary containing the major classes of relationship terms between miRNAs and their target genes/proteins was also developed. A tri-occurrence based approach was used to extract the MTIs (co-occurring with a relation term) at the sentence level.

Using the above-mentioned dictionaries, our group previously harvested AD specific PPIs from MEDLINE abstracts and full text articles [69]. Here we used the interaction terms compiled by Thomas et al. [70]. A state-of-the-art machine learning based approach [71] was applied to retain true pairs of PPIs in a given sentence. Both approaches have been optimized for recall. Hence, the obtained relations have been manually filtered for false positives. After manual inspection, 339 PPIs for 301 proteins and 99 MTIs for 36 microRNAs that are specific to AD were obtained. Articles published in languages other than English could lead to increased information content, however a dedicated approach to harvest them is needed. Moreover, separate parsers are needed. Thus, for this work we extracted interactions from the biomedical literature in English.

#### AD gene expression data

A standard approach to test any generated hypothesis is to assess the gene expression of the involved candidates between affected and healthy patients or in the absence of human data we fall back to animal models or derived cell cultures [72–75]. High-throughput technologies such as microarray, RNA-seq provide potential to measure gene expression simultaneously for different experimental/biological conditions. These studies are assembled in widely adopted public archives: The NCBI Gene Expression Omnibus (GEO) [76] and ArrayExpress [77].

For querying AD-specific gene expression data, we used previously developed database, NeuroTransDB [78], which contains highly curated meta-data information for eligible AD studies. It assembles studies from public resources namely, GEO and ArrayExpress, using a keyword based search approach. Among the 45 prioritized AD human studies, we filtered for microarray studies that measure gene expression in brain tissue extracted from both AD and healthy patients. In addition, availability of raw data was a mandate for applying uniform pre-processing. In total, we obtained eight microarray studies to be integrated in *NeuroRDF*: GSE12685, GSE1297, GSE28146, GSE5281, E-MEXP-2280, GSE44768, GSE44770, and GSE44771.

To assess the quality of the arrays we applied ArrayQualityMetrics [79] package. The selected studies (independent of the platform type) were pre-processed using Bioconductor (Version 3.0) packages in R [80], by applying similar methods for maintaining consistency by reducing variance. All studies conducted on Affymetrix chips were normalized by robust multi-array average method (*rma*) [81]. Similarly, package *limma* [82] was applied on Rosetta/Merck Human 44 k 1.1 microarray chip. All the chips were normalized for background correction and quantile normalization. The normalized intensity values were log2-transformed and duplicate probes were averaged. To identify the differentially expressed genes between healthy and Alzheimer’s patients we used *limma* package by applying Benjamini and Hochberg's method to control for false discovery rate (adjusted *p*-value ≤ 0.05).

### Data curation

Although the current text-mining methods have started to leverage expert curators to extract PPIs, MTIs, etc. from text, the extracted information are still prone to false positives [83]. Moreover, it is not straightforward to use these systems for retrieval of context-specific triples due to technological limitations [84]. Hence, the meticulousness of the identified triples to occur in a certain cell type, disease state, or events captured in AD-specific documents is not guaranteed. Thus, the need for manual verification is unavoidable, especially when considering the full text articles. The previously published test corpus used for evaluating the constructed AD PPI network contained AD-specific PPIs extracted by the machine learning approach from 200 full text articles [69]. Manual inspection by the authors resulted in retaining PPIs from 38 articles that are truly specific to AD, thus discarding 81 % of the originally retrieved articles. Similarly, we retained only 68 abstracts from 250 articles (27 %) that were retrieved using our tri-occurrence based approach for AD MTIs [65]. Thus, we can conclude that only about 20–30 % of the (relation extraction based) extracted PPIs and MTIs are truly relevant to AD, pointing out the need of manual curation.

Similarly, in our recent publication [78], we have highlighted the key issues related to retrieval and reusability of the datasets from public transcriptomics archives, such as GEO and ArrayExpress. We showed that a simple keyword based search not necessarily asserts the specificity of the retrieved datasets to the queried disease or organism. When manually inspected, we reported nearly 20 % of these retrieved studies to be irrelevant for AD query. In addition, basic metadata annotations such as age, gender, etc., which strongly contributes to the differential estimates, were observed to be incomplete. Brazma et al. [85] had earlier reported that not all the data submitted to GEO or ArrayExpress are MIAME compliant [86]. We additionally noticed these missing annotations being scattered as unstructured prose in database webpages, publications, supplementary material, figures, etc., leading to a steep increase in the needed curation effort. Although the published research articles are rich in annotations, a large number of experiments have missing citations [87], which have to be added manually. Moreover, inconsistencies between the information stored in the archives and in the associated publications were also noted. On an average, about 30 min to 2 h of curation effort was needed to retrieve pertinent information for a single dataset. The outcome of this work resulted in a highly curated metadata database, NeuroTransDB, which is used in this work for extracting relevant AD gene expression studies.

### Generation of RDF models

#### RDF data model

RDF allows the generation of models for processed data that exchanges information on the Web [82]. The RDF data model stores all the relationships between different entities as triples (subject-predicate-object). In RDF terminology, the subject, the predicate and the object are known as resources and are represented by a unique “Uniform Resource Identifiers (URIs)" in order to support global data exchange. Literals are constant values such as numbers and strings mapped to the resources. Literals can only be used as objects but never as subjects or predicates.

#### RDF schemas

We constructed the RDF schemata by abiding the standard RDF graph notation where an ellipse represents Resource, an arrow for Property, and rectangle for Literal. In all the RDF schemas, we have maintained a common resource representation for the “Gene" namespace adapted from Bio2RDF that maps to the NCBI gene database. For the namespaces with no available ontologies, we created an internal namespace, called “SCAI". Some of the properties were described using URIs from Dubin Core Metadata Element [88].

**Fig. 2:**
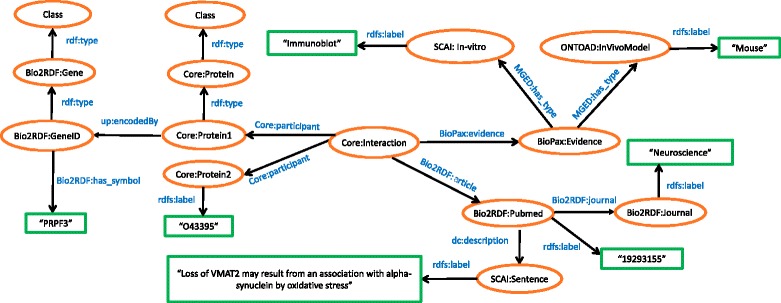
Schematic representation of the Diseased PPIs in RDF. The figure describes AD specific PPI interactions along with supporting evidence mined from literature

**Fig. 3:**
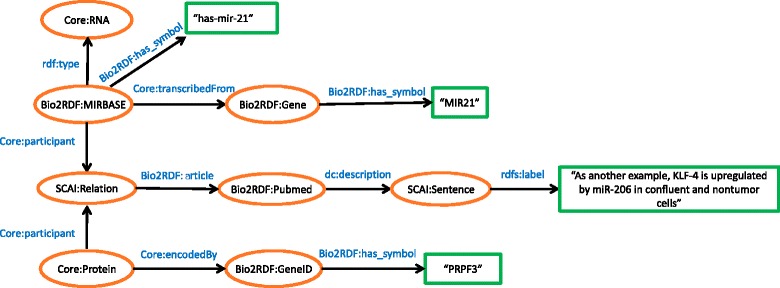
Schematic representation of MiRNA-target interactions in RDF. The figure encapsulates miRNA mentions along with their corresponding gene identifier from literature

Four separate schemas (for each data resource) have been generated that are centered on genes for interoperability, associating each gene product to its official gene symbol. In the AD PPI schema (see Fig. 2), proteins and their interactions were represented using the Uniprot Core Ontology [89]. Supporting literature evidence were adapted to URIs from Bio2RDF namespaces. The article resource was linked to its PubMed ID, sentence in which the interaction has been mentioned, and the associated journal. Experimental evidence that validates the given interaction (if any) were mapped to BioPax [90], MGED [91], ONTOAD [92], and SCAI namespaces. In the MTI models (see Fig. 3), literature, genes, and proteins namespaces were adapted similarly to the PPIs. To represent the miRNAs, we applied the Bio2RDF namespace that links it to miRBase database [93].

**Fig. 4:**
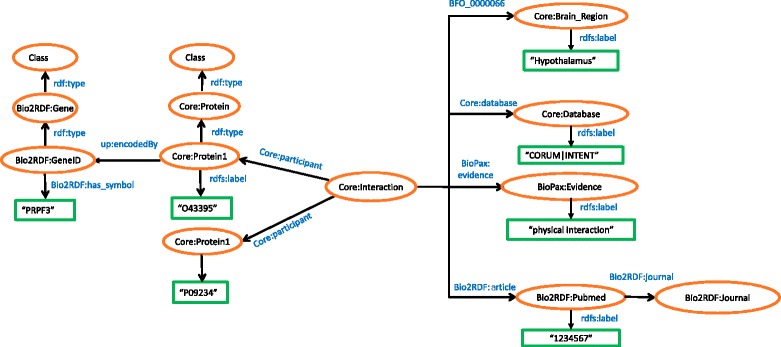
Schematic representation of Healthy PPIs in RDF. The figure represents PPIs of healthy subjects extracted from literature and PPI specific databases. The schema also contains meta-information about these PPIs

For the PPI schema encoding the healthy state, as seen in Fig. 4, we used the same ontologies as in case of AD PPI. Additional interaction evidence such as brain region, reference database, experimental evidence, and literature information were described using Core, BioPax, and Bio2RDF namespaces.

**Fig. 5:**
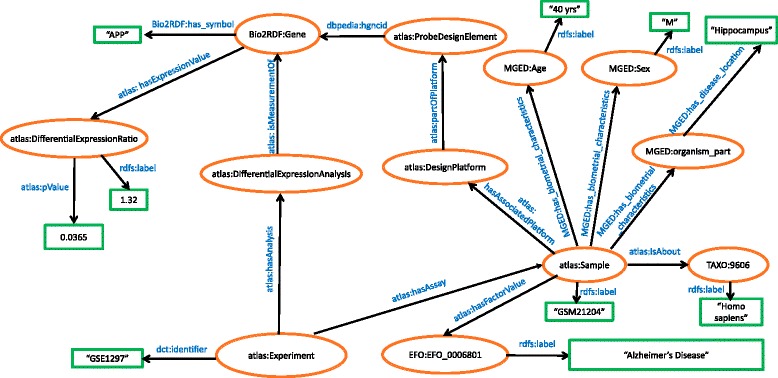
Schematic representation of Gene Expression Data in RDF. This figure represents gene expression data obtained from public resources such as GEO and ArrayExpress

The microarray schema has two branches that are linked to the experiment: sample details and differential expression analysis. The majority of the resources and properties are linked to URIs from EBI's Atlas (atlas) [94] and MGED [91] namespaces, cf. Fig. 5. Gene expression experiments could contain several samples that are measured in different conditions. A detailed description of each sample is needed for accurate analysis. Thus, we associated each sample to its meta-data annotations, namely age, gender, organism, organism part, platform, and phenotype. Organism under investigation is mapped to NCBI Taxonomy URIs [95]. The factor value of each sample, i.e., the phenotype information, is described using the EFO ontology [96]. Each platform array is made up of multiple probes that may represent a gene. To be able to retain the expression values for individual probes, we linked the probe ID resource to platform. However, for better reasoning, quantitative values retrieved from statistical analysis are linked to genes and not to probes. The meta-analysis results, derived from *limma* [82], such as differential expression value of a gene and its associated p-value are all linked to the gene symbols.

#### Construction, validation and storage of RDF models

We modeled all the triples (represented in the schemas) using the Apache Jena API [97]. Resources, and Properties as Java classes were created from the ontologies using the corresponding in-built methods in the API and with the help of Schemagen [98].

In order to check for the correctness of our generated RDF models, we made use of the online service RDF validator [99]. By using such a service, we verified the models using their graph and triples representation.

Triple stores, such as Virtuoso [100], provides an opportunity to store individual or integrated RDF models in one endpoint. Taking advantage of this, we stored all the generated RDF models as individual graphs in a single Virtuoso instance. Using common URIs (e.g., “Gene" identifier) as the connecting link between these models, it is possible to traverse through them integratively.

### Data mining and analysis

In RDF, all the stored triples are accessible using a common query language, SPARQL Protocol and RDF Query Language (SPARQL) [101]. We generated a Java library with embedded SPARQL queries to ask our endpoint and the underlying networks biologically relevant questions. Queries were generated from individual models, which were further integrated as nested queries to traverse different graphs. Each query uses the common Gene URI namespace (which is common across all models) to pass on the results used to the next nested query. One possibility to visualize the query results is the SemScape Cytoscape [102], to represent the return values as (sub-) graphs again.

## Results and discussions

NeuroRDF covers a wide range of curated AD related data resources, stored as four separate RDF models in a single Virtuoso endpoint. It tries to address the main concepts (complementary) that contributes significantly to unraveling AD pathology.

### Differentially expressed genes

For the eight selected microarray datasets, gene expression analysis was performed between healthy and diseased patients. Among these, GSE1297, GSE28146, and E-MEXP-2280 resulted in no differential genes for adjusted p-value cutoff 0.05. From the remaining studies, only genes that exhibited a log2 fold change of > 1.5 were selected for analysis. In total, GSE5281 resulted in 4,278 genes under p-value cutoff and 2 up-, and 48 down-regulated genes for the defined fold change cutoff. Similarly, GSE44770 provided 254 differentially expressed genes, among which 16 up- and 11 down-regulated were selected further. In case of GSE44771, we obtained 335 differential genes that contain 11 up and 11 down-regulated genes that show > 1.5 log2 fold change. For both, GSE12685 and GSE44768, we obtained 1 and 51 genes under the p-value cut-off. However, there were no genes that had log2 fold change of >1.5. The list of all the differentially expressed genes that were selected for further analysis is provided in Additional file 1.

### RDF models

Table 1 summarizes the content of the generated triple store by providing some statistics of all integrated networks. In total, there are 8353 unique triples in AD PPI, 1,204,194, 667 unique triples in Healthy PPI, and 20,454 unique triples in gene expression RDF models (Additional file 2). The number of unique predicates (relations) for AD and healthy PPIs are 11, whereas for MTI there are 5 and the gene expression model consists of 16. The number of entities present in these models range from 300 to 78,852 (cf. Table 1). In case of the gene expression data, to avoid large triples we excluded the gene expression values of individual probes and included information only related to differential expression. Uploading and querying these models was not computationally expensive due to lower set of predicates and relatively small file size.

**Table 1:** Statistics of generated RDF models stored in Virtuoso endpoint

Models	No. of triples	No. of entries	No. of properties	Size (mb)
Alzheimer’s disease PPI	8353	19900	11	0.894
Healthy State PPI	1204194	78852	11	99.102
MTI	667	300	5	0.095
Microarray	20454	9477	16	303.5

## Prioritization of AD candidates

To illustrate the potential of NeuroRDF approach and to determine novel AD candidates from the high quality integrated data, we exploit the underlying biological association between the different data resources and identify the previously unknown information.

**Fig. 6:**
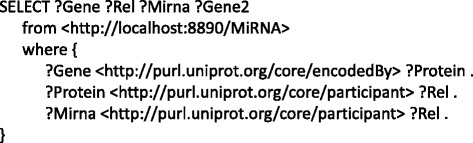
Example SPARQL query for information retrieval from NeuroRDF. SPARQL query as seen in the figure retrieves the miRNAs for a given gene

Our prioritization criteria was based on the notion that every data resource brings with it a piece of missing biological information which is needed to understand the mechanism of a certain candidate. We tried to associate this distributed information by systematically addressing the following questions:

1. Whether candidates in the diseased network tend to be associated with normal physiology. If yes, what are the common players that could help us in the differential estimates (called as causal candidates);
2. Which microRNAs regulate the selected causal candidates that could give insights into their post-transcriptional dysregulation;
3. Have any of the selected causal candidates assessed for their level of differential expression in an unbiased data source (e. g., gene expression data);
4. How strong is the influence of the neighboring genes on the casual candidates. This is based on the assumption that strong candidates tend be surrounded by dysregulated genes and have an influence on the candidate itself;
5. Is there any functional relatedness between the causal candidates and their neighbors;

To answer these questions, we generated a set of SPARQL queries. Figure 6 is an example SPARQL query syntax used to obtain miRNAs that regulate the genes in the AD networks. Similar querying has been applied to build a system of faceted searches for the above described questions. Firstly, we identified common genes between the healthy and AD PPI networks. This query resulted in 230 intersecting genes. Looking into the MTIs, we found 13 of these genes to be regulated by at least one microRNA (cf. Table 2). Among these 13 genes, 9 were observed to be differentially expressed: APP, BACE1, ADAM10, IL1B, MAPK3, DLG4, LRP1 PTGS2, and TGFB1. Except for APLP2, and IL6, all the other genes contained differentially expressed neighbors either in AD or in healthy PPIs. There were no miRNAs that were common to these 13 genes.

**Table 2:** Prioritized AD candidate genes

Intersected genes between healthy and AD PPI	MiRNAs	Differentially expressed neighbors		Number of literature articles for intersected genes
		Healthy PPI	AD PPI	
APP	MIR101-1,	ADAM10,	TGFB1,	
	MIR106A,	MAPT,	BACE1,	
	MIR106B,	MIF,	LRP1	
	MIR124-1,	BACE1,		24550
	MIR137,	LRP1		
	MIR153-1,			
	MIR181-C,			
	MIR29A,			
	MIR520C,			
	MIR19-1			
BACE1	MIR107,			
	MIR124-1,		APP,	
	MIR145,	APP	LRP1	1883
	MIR298,			
	MIR29A,			
	MIR29B1,			
	MIR328,			
	MIR9-1			
ADAM10	MIR451,			
	MIR144,			
	MIR1306,	APP	-	231
	MIR107,			
	MIR103			
IL1B	MIR146A,			
	MIR155	PTGS2	-	1099
MAPK3	MIR15A,	-	STMN2,	276
	MIR155		JUN	
MAPT	MIR16-1,	APP	TUBA4A	3367
	MIR132			
APLP2	MIR153-1	-	-	134
DLG4	MIR485	-	LRP1	151
IL6	MIR27B	-	-	748
JUN	MIR144	-	STAT4,	142
			MAPK3	
LRP1	MIR205	APP	DLG4,	305
			APP,	
			BACE1	
PTGS2	MIR146A	IL1B	-	474
TGFB1	MIR155	-	APP	276

This table summarizes the literature based evidences of intersected genes between healthy and AD PPI and their corresponding miRNAs and differentially expressed genes

**Fig. 7:**
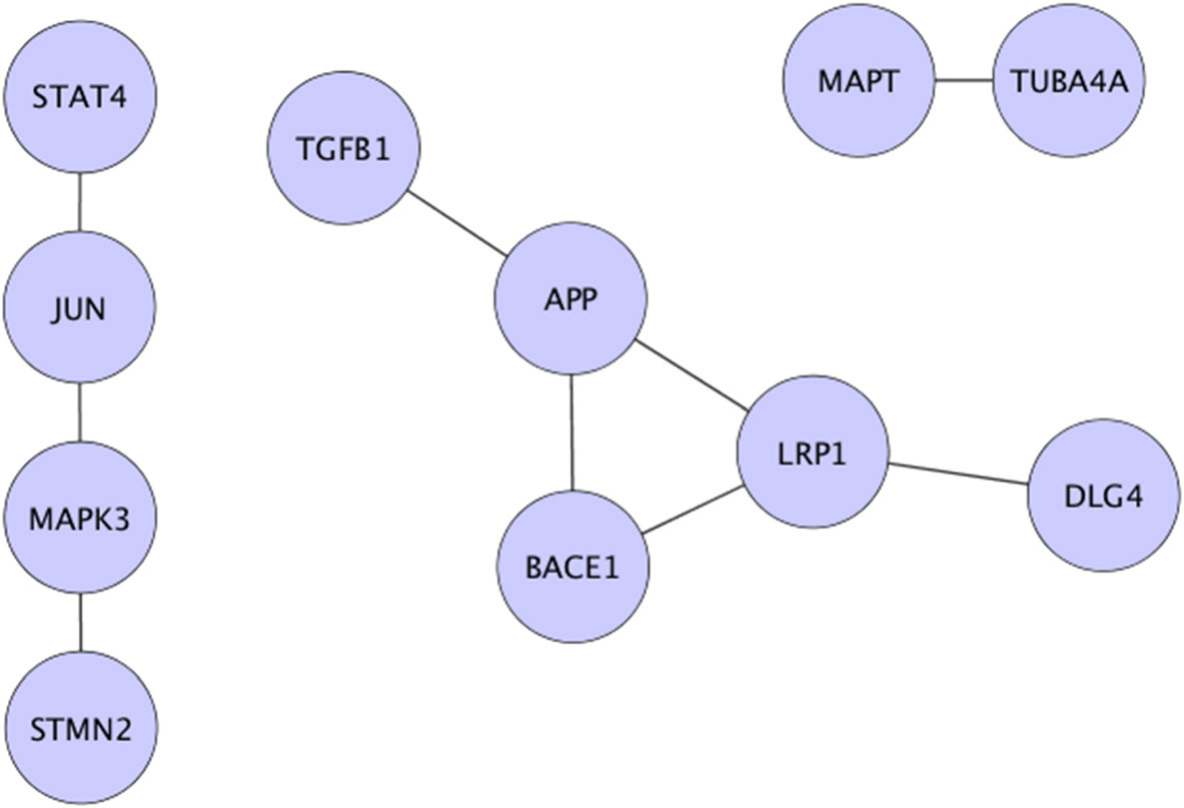
Extracted sub-networks from AD PPIs network. This figure symbolizes the diseased sub-graphs that were generated using prioritized candidates and their differentially expressed neighbors

**Fig. 8:**
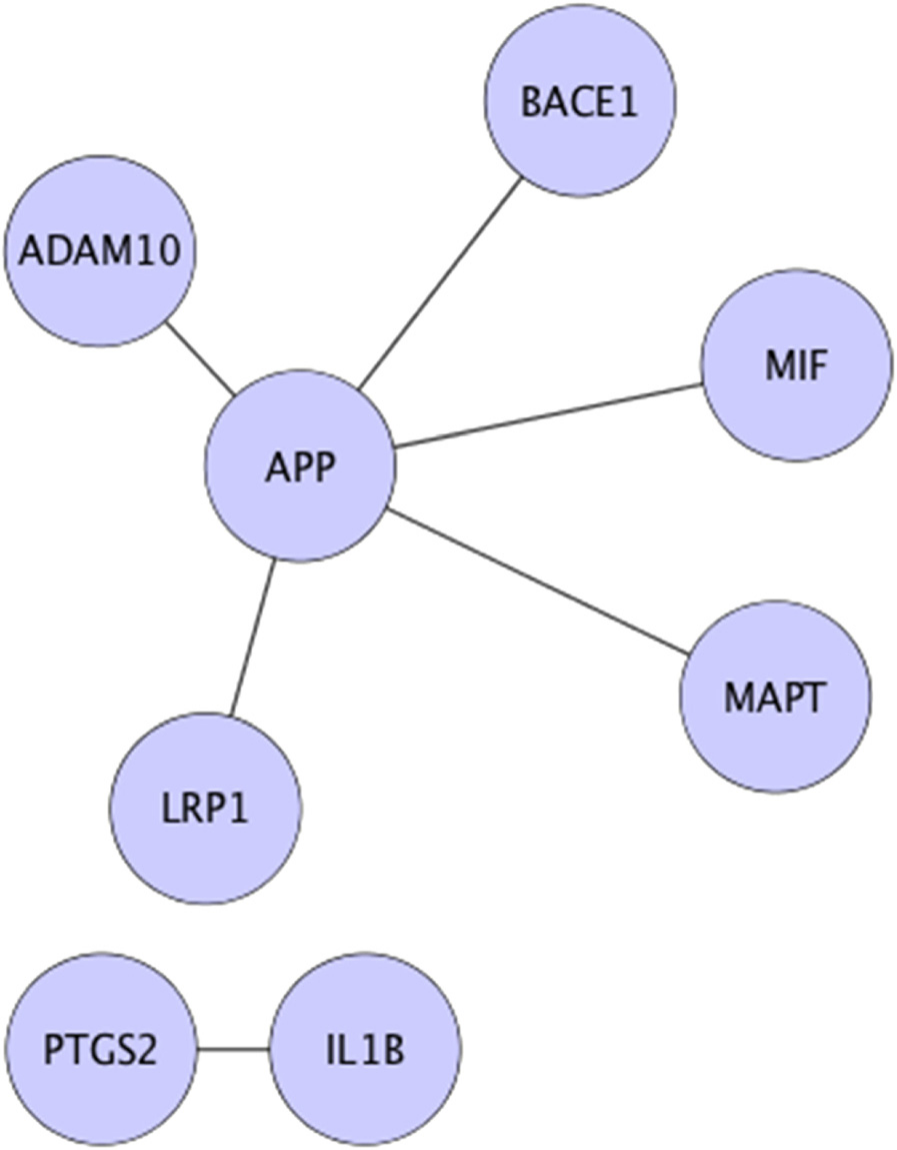
Extracted sub-networks from healthy PPIs network. This figure symbolizes the healthy sub-graphs that were generated using prioritized candidates and their differentially expressed neighbors

Sub-networks from the AD and healthy PPIs were extracted to investigate the prioritized candidates (see Figs. 7 and 8). As observed from Fig. 8, for healthy PPIs there was one larger sub-network (containing APP, ADAM10, BACE1, MIF, MAPT, and LRP1) and a smaller one containing two genes (PTGS2, and IL1B). On the other hand, for diseased PPIs in Fig. 7, there were two large sub-networks containing four (STAT4, JUN, MAPK3, and STMN2) and five genes (APP, LRP1, BACE1, DLG4, and TGFB1). The third sub-network was made up of two genes (MAPT, and TUBA4A). Among the prioritized candidates, APLP2 and IL6 had no common links to other prioritized candidates. Thus, they were discarded for further analysis.

### Relevance of prioritized AD candidates

The remarkability of complementing wet lab research using the predictability and reproducibility of measured outcomes is one of the core reasons why researchers are more inclined to the field of bioinformatics. Therefore, in silico validation of predicted candidates for its relevancy is of utmost importance. In this direction, we pinpoint the relevance of our prioritized candidates through a literature survey.

#### AD established candidates

Although there are no FDA approved biomarkers for AD, researchers focus on some of the key candidates that are hypothesized to be involved in AD. In the current NDD research practice, APP has been established as the widely used biomarker candidate. The classical pathological hallmark of AD is formation of amyloid-beta aggregates (leading to plaques) in brain. This is reported to be caused by faulty proteolytic processing of APP that releases amyloid-beta [103]. Another hallmark of AD is tau pathology (MAPT gene), regulated by amyloid-beta. Hyperphosphorylation of tau causes accumulation of neurofibrillary tangles due to the disrupted functioning of axonal transport [5]. However, it is also interesting to note the paradigm shift in AD research due to recently failed drug trails that focused mostly around these hypotheses [2]. Nevertheless, several neuroscientists still believe in the potential of APP and the tau hypothesis for elucidation of the underlying pathomechanism. As observed from our generated sub-networks, our largest sub-network was established around the APP gene.

When compared to APP, BACE1 has not been so frequently studied. However these genes often fall into the "most interesting gene zone" as far as AD is concerned since it is involved in the formation of amyloid-beta. BACE1 is the major enzyme (beta secretase) involved in the cleaving of APP at beta site and generating soluble amyloid-beta [104]. However, increased BACE1 activity has been reported to be associated with amyloid-beta aggregation in AD patients [105]. Bu et al. have detailed out the evidence that LRP1 is a receptor for APOE, a contributing factor to AD [106]. Furthermore, in 1993, Strittmatter, Roses and colleagues [107] have identified APOE4 as the major risk for late-onset AD. TGFB1 polymorphism has been widely associated with an increased risk of late-onset AD. Deficiency in TGFB1 signaling leads to neurofibrillary tangle formation increasing the advancement of mild cognitive impairment patients to AD, by increasing the depressive symptoms [108]. DLG4 is a post-synaptic scaffolding protein that interacts with postsynaptic receptors such as NMDA receptors for efficient postsynaptic response [109]. However, its impairment has largely contributed to the synaptic degeneration in AD. Mutations in ADAM10 gene have been associated to late-onset AD. ADAM10 enzyme has alpha-secretase activity to cleave amyloid-beta, however BACE1 competes with ADAM10 for cleavage. Thus, its decreased expression has been implicated in AD pathogenesis [110].

#### AD emerging candidates

**Fig. 9:**
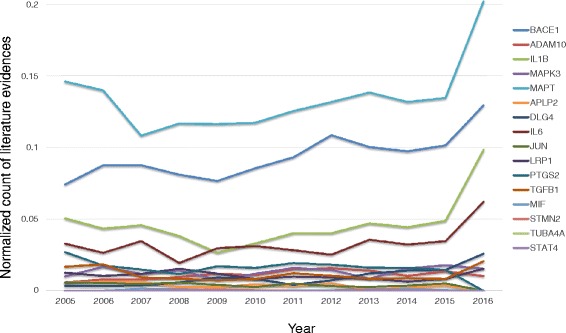
Statistics of the literature evidence for emerging candidate genes in the speculated sub-networks

To identify emerging knowledge in the context of AD, we performed an individual gene analysis using SCAIView for publications in PubMed. Here, we measured the co-occurrence of the causal genes (including its differential neighbors) and AD over a period of last 10 years, see Fig. 9. Since the number of articles for the APP gene was relatively too high each year, we normalized the number of literature evidence of other candidates using the APP gene's article count for that year. Hence, the normalized range for the literature distribution is between 0 and 1, where 1 is the highest number of articles for that year (the APP gene). Please refer to Additional file 3 for details of the literature counts. Inspecting literature evidence, we found that all the prioritized causal candidates have been studied in the context of AD. Moreover, among their differentially expressed neighbors, STMN2 (8 articles), MAPK4 (1 article), TUBA4A (2 articles), and MIF (15 articles) contained fewer articles related to AD. Among these genes, STMN2 and MIF have been recently studied in the context of AD, whereas, MAPK4, STMN2, and TUBA4A were implicated in AD nearly two decades before but failed to establish as robust biomarker candidates.

### MIF's role in AD

Macrophage Migration Inhibitory Factor (MIF) has for long been known to participate in tumor proliferation due to its pro-inflammatory cytokine functionality [111]. In general, MIF acts as a key regulator of inflammatory activities such as innate and adaptive immunity [112]. Apart from that, it is also known to play a significant role as an anti-apoptotic factor of neutrophils as well as macrophages [113].

The MIF gene has been well studied in cancer and inflammation. However, recent studies are emerging around a plausible role of MIF in neurodegenerative diseases, in particular AD. Moreover, Flex et al. [114] have earlier reported that MIF polymorphisms are not linked to AD, but confirmed its complex immune and inflammatory activities. Although, APP and tau have been associated to play a key role in the pathophysiology of AD, many researchers strongly believe in the role of inflammatory processes subsidizing to the pathology of AD. This stems from the fact that activated microglial cells discharge immunoregulatory cytokines which result in various side-effects such as neuronal dysfunction and inhibition of hippocampal neurogenesis [115]. MIF is one such pro-inflammatory cytokine which is known to bind with amyloid-beta protein and enhance the plaque removal and neuronal debris from the brain during normal conditions [116]. Also, MIF has been identified to play a role in neuronal survival by inhibiting the activation of ERK-1/MAP kinases [117] (regulatory role in cell proliferation and glucocorticoid action) as well as its ability to surpass the p53 mediated apoptosis [118]. Although, the precise molecular function of MIF in the context of AD is unknown, it is known to play a role in inflammatory processes around the plaque formation. MIF is also highly expressed in the neurons of rat hippocampus, one of the primary regions to be affected by AD [117]. Bryan et al. [119] also report on the abnormal expression of MIF in both microglia and in the hippocampal neurons in human. This all makes MIF a plausible biomarker for inflammatory responses in AD.

## Conclusion

NeuroRDF approach has been designed to identify new knowledge through semantic mining. The proposed integrative approach takes advantage of the RDF technology to integrate well-curated data from various sources within a specific indication area. From our perspective, it is necessary to focus on one indication or at least a group of indications to build such a knowledge base for precise modeling and analysis due to the high curation effort one has to spend in order to reach the necessary details. We showed how to harmonize three major heterogeneous resources (databases, gene expression data, and literature) used in the research area to generate hypotheses for underlying disease mechanisms. This approach supports identification of novel insights without compromising over quality. Furthermore, new data resources can be included without altering the overall framework. The usage of well-accepted ontologies provides the advantage for further integration of external resources and databases (e.g., federated queries). Using such an approach, we were able to prioritize MIF gene as an emerging candidate due to its role in inflammatory processes implicated in AD pathogenesis.

The advantage of using an RDF schema is that it is highly supportive for data interoperability. Although this work represents the usage of the RDF schema specific for AD, we have also extended the same to other disease models such as Parkinson's and Epilepsy. However, the curated data and the generated hypothesis for these two diseases will be released in future under the terms of a Neuroallianz agreement [120]. Also, these resources are constantly kept up-to-date as they are transferred to various upcoming projects such as AETIONOMY [121].

## Additional files

Additional file 1: List of differentially expressed genes. This file contains the list of differentially expressed genes (for each dataset used) that fall under the adjusted p-value cutoff of 0.05. The differential expression analysis was performed using *limma* package in R statistical environment. The file is provided in an Excel format. (XLSX 68 kb) Additional file 2: The developed RDF models and the SPARQL queries used are made available at: http://www.scai.fraunhofer.de/en/business-research-areas/bioinformatics/downloads/neurordf.html. (ZIP 178 kb) Additional file 3: Detailed count of literature evidences for prioritized candidates. This file contains the detailed count of number of evidences available for each prioritized candidate for each year since 2005 in context of Alzheimer's disease. These statistics were retrieved using SCAIView knowledge discovery tool (as of 18 May, 2016). (XLSX 35 kb)
